# Supplementary material for: Inter-Day Variation in the Fasting Plasma Lipopolysaccharide Concentration in the Morning Is Associated with Inter-Day Variation in Appetite in Japanese Males: A Short-Term Cohort Study
Source: Metabolites. 2023 Mar 8;13(3):395. doi: 10.3390/metabo13030395 (PMC10053071; doi:10.3390/metabo13030395)
Supplement: Supplementary file 1 [file metabolites-13-00395-s001.zip › metabolites-2252627-supplementary.pdf]

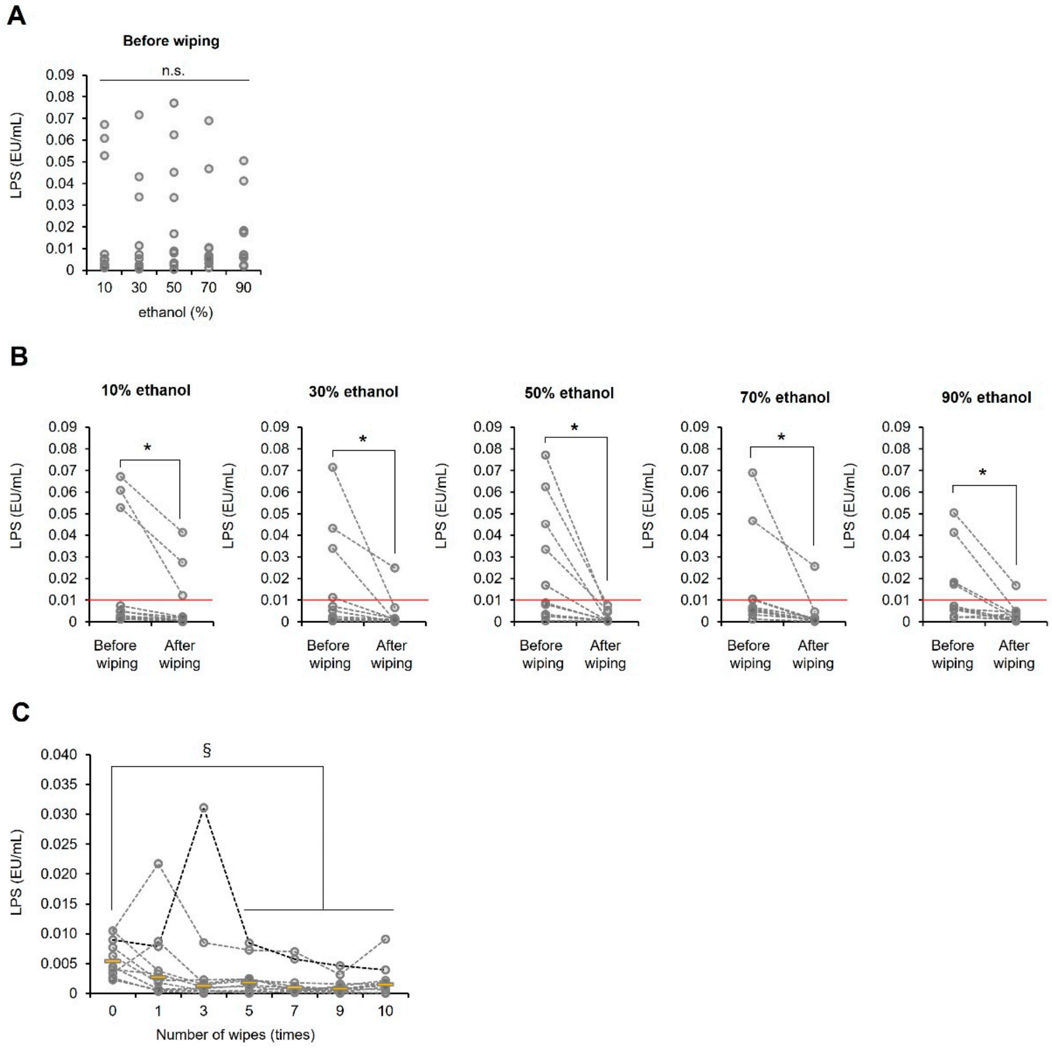

Figure S1. Ethanol concentration and wiping times of ethanol-impregnated cotton for optimal removal of lipopolysaccharide from fingertips. (A, B) In order to allow environmental lipopolysaccharide (LPS) to adhere to the skin, three healthy subjects were asked to perform daily activities with bare hands (e.g., contact with tap water, doorknobs, desks or walls, computer work, etc.). Then, 50 μL of LPS-free distilled water (Otsuka distilled water; Otsuka Pharmaceutical Co., Ltd., Tokyo, Japan) was dropped onto the fingertip of each subject using a micropipette in a hemispherical shape, allowed to stand for 30 seconds, and then the entire amount was collected (sample before wiping). The same finger was wiped with 10% (v/v), 30% (v/v), 50% (v/v), 70% (v/v), or 90% (v/v) ethanol-impregnated cotton, 10 times each, and then 50 μL of LPS-free distilled water was dropped onto the fingertips and then collected as well (post-wipe sample). One type of ethanol-impregnated cotton was used per finger. (A) The LPS concentration in before wiping samples are shown, in which the circles denote the actual measurements. There were no significant differences (n.s.) of the LPS concentrations in before wiping samples (Steel-Dwass test, $P > 0.05$, n = 10) (B) The LPS concentrations before and after wiping in each ethanol impregnated cotton are shown, in which the circles denote the actual measurements and red line indicate cut-off value. Comparison of the LPS concentrations between before and after wiping samples showed a significant decrease in the LPS concentrations at all ethanol concentrations (Wilcoxon signed-rank test, $P < 0.05$, n = 10). However, only when fingertips were wiped with 50% (v/v) ethanol-impregnated cotton, the LPS concentrations of after wiping samples were below the cutoff for all. (C) To examine the optimal number of wipes, fingertips were wiped 10 times with 50% (v/v) ethanol-impregnated cotton. After 0, 1, 3, 5, 7, 9, and 10 wipes, the LPS-free distilled water were dropped onto fingertips and collected as in (A, B) and the LPS concentration in the samples was evaluated. the circles denote the actual measurements and orange line indicate median value. The LPS concentration was significantly decreased after 5 or more wipes compared to before wiping

(Steel test, $P < 0.05$, n = 10).

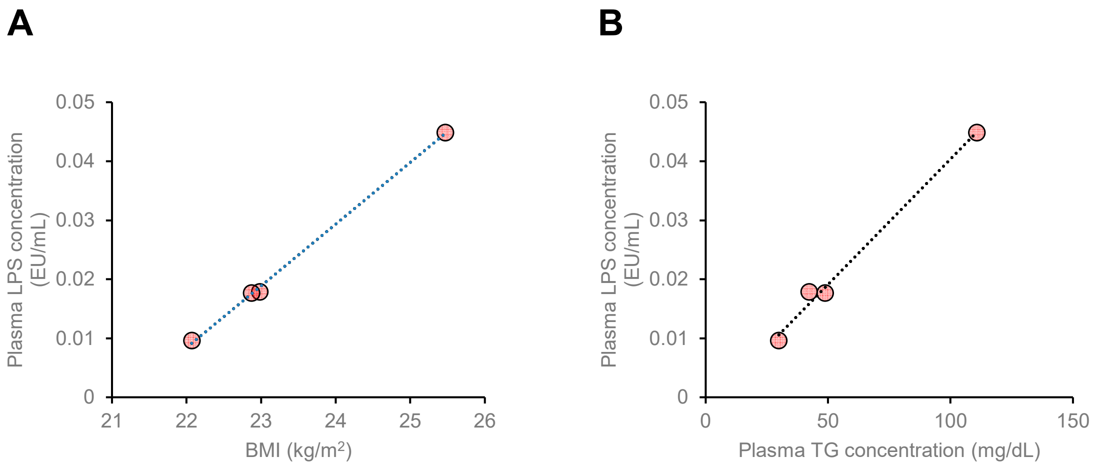

Figure S2. The correlation of fasting plasma lipopolysaccharide concentration with fasting body mass index and fasting plasma triglyceride concentration (actual values). Scatter plots show the correlation between the 10-day mean fasting plasma lipopolysaccharide concentration and the 10-day mean fasting body mass index (BMI) (A) or fasting plasma triglyceride (TG) concentration for each participants (#A to #D). Circles denote actual measurements, and dotted lines represent the regression lines (n = 4).
